# Codon usage in twelve species of *Drosophila*

**DOI:** 10.1186/1471-2148-7-226

**Published:** 2007-11-15

**Authors:** Saverio Vicario, Etsuko N Moriyama, Jeffrey R Powell

**Affiliations:** 1 Department of Ecology and Evolutionary Biology, Yale University, New Haven, CT, 06520-8105, USA; 2 School of Biological Sciences & Plant Science Initiative, University of Nebraska-Lincoln, Lincoln, NE, 68588-0660, USA; 3 Department of Ecology and Evolutionary Biology, Yale University, 21 Sachem Street, ESC Room 172, New Haven, CT, 06520-8105, USA

## Abstract

**Background:**

Codon usage bias (CUB), the uneven use of synonymous codons, is a ubiquitous observation in virtually all organisms examined. The pattern of codon usage is generally similar among closely related species, but differs significantly among distantly related organisms, e.g., bacteria, yeast, and *Drosophila*. Several explanations for CUB have been offered and some have been supported by observations and experiments, although a thorough understanding of the evolutionary forces (random drift, mutation bias, and selection) and their relative importance remains to be determined. The recently available complete genome DNA sequences of twelve phylogenetically defined species of *Drosophila* offer a hitherto unprecedented opportunity to examine these problems. We report here the patterns of codon usage in the twelve species and offer insights on possible evolutionary forces involved.

**Results:**

(1) Codon usage is quite stable across 11/12 of the species: G- and especially C-ending codons are used most frequently, thus defining the preferred codons. (2) The only amino acid that changes in preferred codon is Serine with six species of the *melanogaster *group favoring TCC while the other species, particularly subgenus *Drosophila* species, favor AGC. (3) *D. willistoni *is an exception to these generalizations in having a shifted codon usage for seven amino acids toward A/T in the wobble position. (4) Amino acids differ in their contribution to overall CUB, Leu having the greatest and Asp the least. (5) Among two-fold degenerate amino acids, A/G ending amino acids have more selection on codon usage than T/C ending amino acids. (6) Among the different chromosome arms or elements, genes on the non-recombining element F (dot chromosome) have the least CUB, while genes on the element A (X chromosome) have the most. (7) Introns indicate that mutation bias in all species is approximately 2:1, AT:GC, the opposite of codon usage bias. (8) There is also evidence for some overall regional bias in base composition that may influence codon usage.

**Conclusion:**

Overall, these results suggest that natural selection has acted on codon usage in the genus *Drosophila*, at least often enough to leave a footprint of selection in modern genomes. However, there is evidence in the data that random forces (drift and mutation) have also left patterns in the data, especially in genes under weak selection for codon usage for example genes in regions of low recombination. The documentation of codon usage patterns in each of these twelve genomes also aids in ongoing annotation efforts.

## Background

The genetic code is redundant, i.e., more than one triplet sequence of DNA bases codes for the same amino acid. Thus genes and species may use different sets of codons preferentially, the phenomenon of codon usage bias (CUB). This well documented un-evenness of use of synonymous codons may come about by a variety of evolutionary forces, in particular, mutation bias, selection, and genetic drift. Generally, species have a characteristic pattern of codon usage that holds across most genes in the species; notable exceptions are warm-blooded vertebrates where isochores, large stretches of DNA with high AT or GC content [1], appear to affect synonymous codon usage depending on the characteristic AT/GC content of the isochore in which a gene resides [2]. There is evidence for heterogeneous regional base content in *Drosophila* (discussed later), although clearly not as strong as in mammals and having much less influence on codon usage.

*Drosophila* have been well-studied for patterns and processes that lead to CUB, in particular *D. melanogaster*, and less often, a few other species for which very limited sequence data were available. The newly available complete genome sequences of 12 species of *Drosophila*[3,4] provide a hitherto unattainable insight into CUB variation and evolution in a set of phylogenetically well-defined related taxa. In addition, knowing the particular codon usage patterns of different species allows more accurate identification of protein-coding sequences (i.e., annotation). Here we examine the patterns of codon usage in these 12 species based on reliably identifiable protein-coding genes that were available in November 2006. We identify patterns of preferred codons for each individual amino acid in each species, as well as examine variation among genes, amino acids, and chromosome arms for level or intensity of CUB in a species. Evidence is provided as to the causes of CUB by considering population sizes and mutation bias as assessed by base composition of introns and non-recombining genes.

## Results

### Not all species have the same overall intensity of codon usage bias

**Figure 1:**
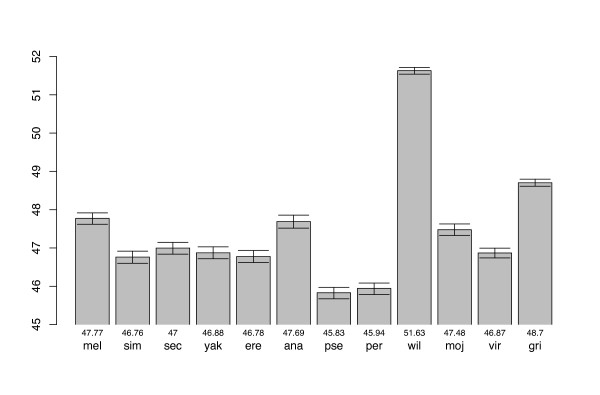
Effective number of codons (ENC) as a measure of overall average codon usage bias in 12 species of Drosophila. 95% confidence bars are shown and the actual means below each bar. Lower ENC indicates greater bias.

We calculated the average effective number of codons [5], ENC, for each species' set of CDSs. We performed a bootstrap randomization test for CDSs with homologue candidates across all species to determine if ENC for different species are statistically significantly different. Figure 1 presents the data. Clearly there is a large range of intensity with Dpse and Dper having the greatest CUB as measured by ENC and Dwil the least. (See Materials and Methods for species abbreviations used in the text.) In the Discussion, we suggest that some of this variation is likely due to differences among species in effective population sizes.

### Identification of preferred codons for each amino acid for each species

**Figure 2:**
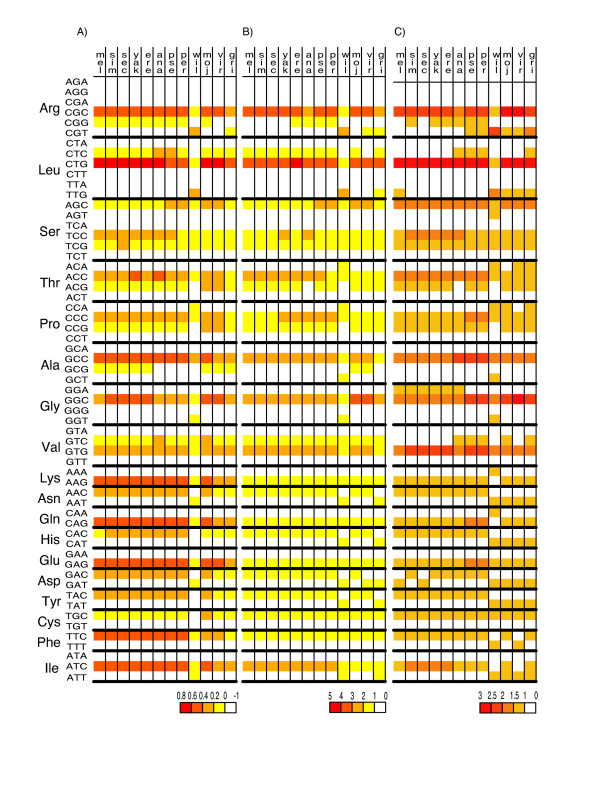
Three methods of identifying preferred codons. In the second column are the 59 codons belonging to the 18 amino acid with more than one codon. The first row indicates the species. The colored shading indicates the values of the relative strength of CUB for the three different statistics used, correlation coefficients for A and B, and RSCU for C. (A) is the correlation between codon usage and overall gene CUB (negative ENC). (B) is the correlation between codon usage and individual amino acid codon usage bias, sENC-X. (C) is the relative synonymous codon usage (RSCU) for the 10% highest biased genes based on ENC. A figure showing RSCU for all CDSs as well as tables with actual values of correlation coefficients and RSCU are included in Additional Files.

Various terms have been used to describe the most used codon for amino acids. The term "optimal" codon is often used, although this term has been co-opted by Duret and Mouchiroud [6] to mean codons associated with high expression of genes, i.e., a mechanism for CUB is assumed. Here we will use the mechanism-neutral term "preferred" codon. Various ways are possible to identify preferred codons and we present three here, which identify very similar sets of preferred codons (Figure 2). Our favored method is Figure 2A, which is to ask: As genes become more biased (uneven) in codon usage, which codon(s) for each amino acid increases in frequency? This is done by simply taking the correlation between overall codon usage bias for each gene (negative ENC) and the use of codons for each amino acid. A related method is to look for correlation between the individual amino acid ENC, sENC-X (see Materials and Methods), and usage of particular codons. Figure 2B presents this correlation between individual amino acid bias and codon usage. A third way of identifying preferred codons is based on the relative synonymous codon usage (RSCU) measure. Figure 2C presents the RSCU for the 10% most highly biased genes in each species as indicated by ENC. The preferred codons identified by these three methods are very similar to one another. But it should be noted that there is an essential difference between the methods used to generate Figures 2A and 2C on the one hand, and Figure 2B on the other. Figures 2A and 2C identify the preferred codons relative to overall level of codon usage bias of entire genes. Figure 2B does this for individual amino acids. Thus at least some of the minor differences may be due to the possibility that evolutionary forces that are dominant on overall codon usage on an entire gene may not always be identical for individual amino acids. This would seem to be the case in Dwil and the subgenus *Drosophila* species in comparing Figures 2A and 2B. While selection may still be acting to provide optimal translation of the entire gene sequence, when selection is weak, those amino acids least sensitive to such selection may not respond and display a codon usage pattern more reflective of mutation bias.

Bootstrap analyses of the data in Figure 2 were done to assess the statistical support for identification of the preferred codon (see Additional File1 and 2, [3]). Overall these analyses indicate strong statistical support of identification of the preferred set of codons for each species. For example, of the 216 highest correlation coefficients in Figure 2A (12 species × 18 redundant amino acids), 209 are supported with a bootstrap value of 95–100%. The other methods give similarly high bootstrap support for the preferred codon (see Additional File 1 and 2).

Two important generalities are clear from Figure 2. First, *the preferred set of codons is quite constant across Drosophila*. Second, *in most cases the preferred codons in all species are those with G and especially C in the third codon position*. That is, generally in *Drosophila*, codon usage is biased toward C/G-ending codons as previously deduced from limited data on fewer species. Exceptions to these two generalities exist, with Dwil being the obvious species with the most exceptions as well as being the species with the least overall CUB (Figure 1). We discuss Dwil in more detail later.

**Figure 3:**
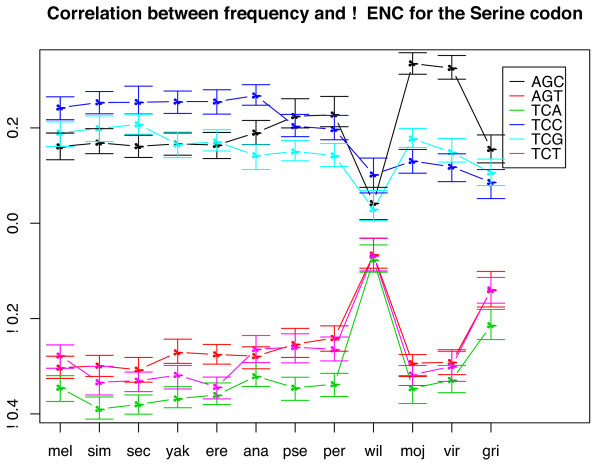
Indication of change in preferred codon for serine. The Y axis is the correlation of usage of each codon with negative ENC of genes, a measure of the relative usage of each codon in each species. Positive correlations indicate favored codons, and negative correlations indicate avoided codons.

Among the 11 species (excluding Dwil), Ser is the only amino acid that actually has a change in preferred codon (Figure 3). TCC is the preferred codon in all members of the *melanogaster *group (Dsim, Dsec, Dmel, Dyak, Dere, and Dana) whereas in the two members of the *obscura *group (Dpse and Dper) AGC is slightly more preferred and in the subgenus *Drosophila* (Dmoj, Dvir and Dgri) AGC is the most preferred codon. In fact, in the subgenus *Drosophila*, TCC becomes the third most preferred codon with TCG rising to the second most preferred.

### Amino acids contribute differently to a gene's CUB

**Figure 4:**
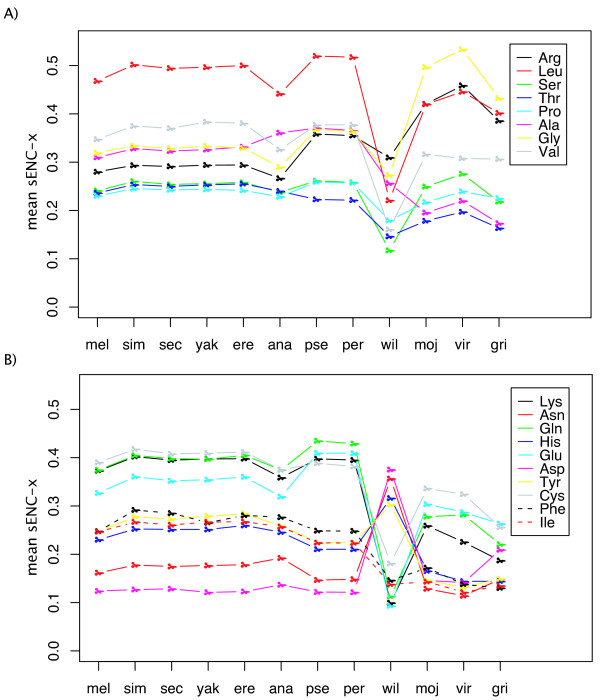
Relative intensity of codon usage bias across the 18 degenerate amino acids in 12 species. The relative intensity is estimated as average sENC-X of each amino acid in each species (Y axis). Amino acids were split to enhance readability with the upper graph (A) containing 4- and 6-fold degenerate amino acids and (B) containing 2- and 3-fold degenerate amino acids.

Here we examine the issue of whether amino acids vary in CUB. Figure 4 presents the results. Note that in this figure we present sENC-x as defined by Moriyama and Powell [7] which scales ENC to have the same range (0–1) regardless of the level of redundancy. In subgenus Sophophora, Leu is clearly the single amino acid that accounts for the greatest CUB of a gene and Asp accounts for the least. This can also be seen in Figure 2 in that the strongest correlations with codon usage are for Leu and the least for Asp. In subgenus *Drosophila* (Dmoj, Dvir and Dgri) Leu still contributes highly to CUB, but Arg and Gly are about equal in high levels of CUB. Similarly, there are differences between the subgenera in which amino acids contribute the least to CUB, with Tyr, His, and Phe being about equally low as Asp in contribution to CUB in subgenus *Drosophila*. Again the uniqueness of Dwil is evident in Figure 4.

### D. willistoni: the outlier

It is evident from Figures 2, 3, 4, that Dwil has a pattern of codon usage very different from all other species. Generally this is seen as an increased usage of A/T-ending codons compared to the 11 other species, resulting in an overall lower level of CUB as measured by ENC (Figure 1). This lowering of overall CUB in Dwil can best be seen in Figure 4 where almost all amino acids produce a blip downwards (toward less bias) in Dwil compared to the other species. In Figure 4, however, note that in Dwil four amino acids, His, Asn, Tyr, and Asp actually increase in sENC-X relative to the other species. These four amino acids are all T/C-ending two-fold degenerate amino acids and are among the weakest in CUB among the 11 other species (Figure 4B); this increase in bias is especially surprising for Asp, which, as noted above, is generally the least or among the least biased amino acids. But it is important to note that the increase in bias of these four amino acids in Dwil is not toward the preferred codon of other species (C-ending) but rather toward T-ending codons. Table 1 summarizes the differences and similarities of Dwil to the other species.

**Table 1:** Summary of preferred codons for each amino acid for 11 species, with differences noted for *D. willistoni*.

**Amino acid**	**Optimal codon**	***D. willistoni***	**Exceptions/notes**
Arg	CGC	CGT	
Leu	CTG	TTG	
Ser	TCC	---	Dpse about equal TCC/AGC preference; Dper, and subgenus Drosophila prefers AGC
Thr	ACC	---	10% highest RSCU identifies ACG in Dmoj and Dvir; ACA in Dgri
Pro	CCC	CCA	10% highest RSCU identifies CCG in Dvir
Ala	GCC	---	
Gly	GGC	GGT	
Val	GTG	GTG/GTC	
Lys	AAG	---	
Asn	AAC	AAT	
Gln	CAG	---	
His	CAC	No preference	10% highest RSCU identifies CAT in Dgri
Glu	GAG	---	
Asp	GAC	GAT	Very weak bias, methods differ somewhat
Tyr	TAC	No preference	10% highest RSCU weakly identifies TAT in subgenus Drosophila
Cys	TGC	No preference	
Phe	TTC	---	10% highest RSCU identifies no preference in subgenus Drosophila
Ile	ATC	ATT	10% highest RCSU weakly identifies ATT in subgenus Drosophila

### Amino acids vary in sensitivity to selection for codon usage

**Figure 5:**
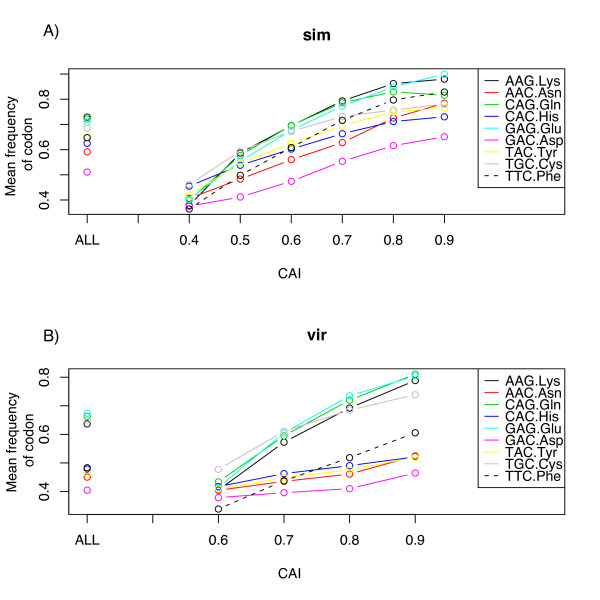
Sensitivity analysis of two-fold degenerate amino acids. Genes were ranked from low to high CAI and binned by 0.1 along the X axis. The frequency of the preferred codon for each amino acid is along the Y axis. Circles to the left are the mean for all CDSs used. Top graph (A) is for Dsim, bottom (B) for Dvir. In both species, Cys, Lys and Gln are the first to respond and Asp the last. See Additional Files for similar graphs for all 12 species.

We also explored whether different amino acids vary in their response to selection for codon usage. If we use the overall CUB of a gene to indicate the level of selection for CUB, we ask whether different amino acids respond differently to selection for overall gene CUB, what we term *sensitivity *to selection for CUB. If, as we discuss later, there is good evidence that the level of CUB increases with increasing levels of gene expression, another way to pose this question is: As proteins increase in level of expression, does codon usage for different amino acids respond differently? We present in Figure 5 results for two species (Dsim and Dvir), one from each subgenus, for two-fold degenerate amino acids; similar graphs for the other ten species are in the Additional File 1 and 2. We confined this analysis to two-fold degenerate amino acids to avoid problems of secondarily or even tertiarily favored codons when more than two codons code for the same amino acid (see Figure 2). The pattern of change in amino acid codon usage is quite consistent between the two species in Figure 5 as well as for the other 10 species (Additional File 1 and 2). Consistent with the patterns of overall contribution to CUB (Figure 4), among two-fold degenerate amino acids, A/G-ending amino acids, especially Lys and Gln are the first to respond to increasing gene CUB (Figure 5), while T/C-ending are less sensitive (with the exception of Cys). Asp, in fact, in Dvir, does not change in frequency of codon usage (Figure 5B). These observations indicate that T/C-ending two-fold degenerate amino acids are under weaker selection compared to A/G-ending two-fold degenerate amino acids, confirming an earlier observation based on much less data [7].

### Mutation bias and codon usage

Mutation bias can affect codon usage especially when selection is weak. In order to assess the pattern and strength of mutation bias, we identify regions of the genome thought to have the least selective constraint, wherein base composition at equilibrium should reflect mutation bias. We identified the intron sequences from the 6,698 CDSs with homologues across all 12 species as described in the Materials and Methods. In order to increase the likelihood of examining selectively neutral DNA sequences, we removed embedded transposable elements as well as 50 bp on each end of the introns in order to avoid constrained splicing signals. We also compared all introns to a subset of introns between 100 bp and 2000 bp (after removal of 50 bp at each end) in order to decrease the probability of including unidentified embedded CDS and control elements more likely to be present in very long introns.

Table 2 summarizes the intron base composition data with further details in the Additional File 1 and 2. Altogether about 20,000 introns representing ten to 30 million bps were identified in the 6698 homologous CDSs, thus providing a very large sample size; trimming the data to introns between 100 and 2000 bp (after removing 50 bps at termini) still provided more than 3,000 introns for each species.

**Table 2:** Base composition (in percent GC) of introns and at the third position of four-fold degenerate amino acids.

	Dmel	Dsim	Dsec	Dyak	Dere	Dana	Dpse	Dper	Dwil	Dmoj	Dvir	Dgri
**All 6,698 homologues**												
No. introns	24960	19890	20742	20768	20716	20826	20492	20467	20535	20784	20772	20876
Total intron length (Mb)	27.9	12.0	12.2	13.3	12.8	14.4	12.7	13.4	18.2	17.0	16.5	15.4
Average intron length (bp)	1119	1546	1520	1637	1582	1991	2075	2107	2565	2236	2120	1754
No. introns: 100/2000^a^	8769	4505	4606	4700	4698	4227	3460	3509	3860	4472	4769	5324
Intron GC% (Wt.)^b^	40.3	41.1	41.0	40.8	41.4	40.8	44.1	44.3	35.1	37.3	38.6	36.8
Intron GC% (Ave.)^c^	37.0	38.5	38.2	37.7	38.6	38.2	43.3	43.9	34.2	35.5	37.3	33.7
Intron GC% (Ave. 100/2000)^d^	37.6	37.9	37.5	37.2	37.9	37.5	42.5	43.0	33.5	34.5	36.5	32.7
GC% 4-fold AA^e^	66.8	68.3	67.9	68.1	68.4	65.8	69.7	69.5	51.0	64.2	66.3	62.8
**33 F homologues**												
Intron GC% (Wt.)^b^	32.1 (31.3)^f^	34.0	32.3	33.3	32.6	39.4	35.4	35.9	35.8	35.8	34.2	30.2
Intron GC% (Ave.)^c^	28.8 (28.4)^f^	29.0	28.3	28.5	27.9	28.8	31.3	31.1	34.1	31.2	30.6	26.9
Intron GC% (Ave 100/2000)^d^	29.8 (29.8)^f^	32.7	31.4	32.0	30.5	30.6	33.8	34.2	33.3	33.9	32.9	26.5
GC% 4-fold AA^e^	37.9	37.8	37.5	37.5	37.5	34.7	43.7	43.3	44.9	43.4	50.8	53.4

^a^Subset of introns between 100 and 2000 bp after removing 50 bp at each end and possible transposable elements.

^b^Weighted average %GC from cumulative sequence (all introns concatenated).

^c^Unweighted average %GC from all introns.

^d ^Unweighted average %GC only from introns between 100 and 2000 bp after removing 50 bp at each end and transposable elements.

^e^GC% of third position of four-fold degenerate amino acid codons.

^f^GC% from all 79 genes known to be on the F element in Dmel that have introns are shown in parentheses.

We present two ways of summarizing the intron base composition data, a weighted mean provided by simply concatenating all introns and calculating base composition, and an unweighted average counting each intron equally regardless of size. Regardless of ways of calculating base content of introns, it is clear that introns are relatively high in A/T, between 59 and 65% across all 12 species. This implies that for all 12 species, *contrary to codon usage bias, mutation bias is toward A/T*. This observation is consistent with the previous observation based on fewer genes mainly from *D. melanogaster *[e.g., [8,9]] and is now shown to be more general for the genus *Drosophila*. (For reasons that will become clear in the next section, we separated genes on the fourth chromosome of Dmel in Table 2.)

Also note that when the data are trimmed to avoid very large introns (>2000 bp), the A/T content consistently increases by about one percent across all species (Table 2), implying that there may be some small degree of unidentified embedded CDSs or control elements in the longer introns. This is consistent with Comeron and Kreitman [10] indicating that higher recombination rate expected in genes with longer introns enhances selection on base composition.

### Non-recombining genes have different codon usage

Most species of *Drosophila* have a "dot" chromosome, the 4^th ^in Dmel, also known as element F [11,12]. Of particular interest is the fact that genes on this chromosome very rarely recombine compared to all the other chromosomes [13,14]. Among the 6,698 CDSs homologous across all 12 species, 33 are known to be on the F element in Dmel, and by chromosome arm homology, may be assumed to be on this element in the other species. Among the 12 *Drosophila* species with completed genome sequences, Dwil is again different in this regard in lacking a dot chromosome [15]. There is good evidence that in Dwil the genes on the F element have been incorporated into one of the other elements, in particular element E [16]. The importance of comparing non-recombining genes to recombining genes is that the effectiveness of selection at individual loci (nucleotide sites in this case) is positively correlated with recombination rate, the well-known Hill-Robertson effect [17]. Codon usage bias has been shown to positively correlate with rate of recombination in *Drosophila*[18-22] and that genes on the (nearly) non-recombining dot chromosome of Dmel are particularly low in codon usage bias [18,23].

**Figure 6:**
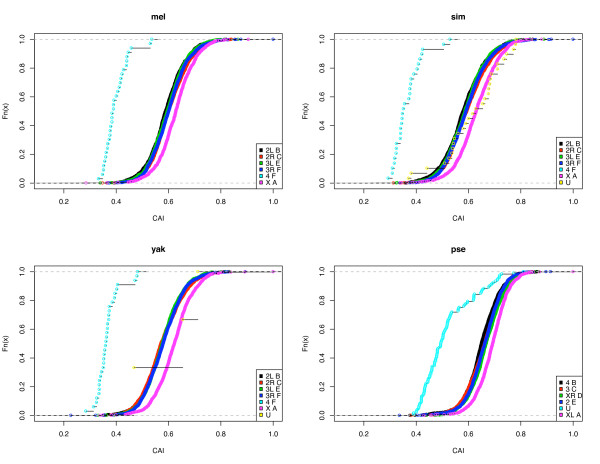
Cumulative distribution of CAI for each chromosome arm. In the lower right of each pane are the chromosomes arm and the Muller/Sturtevant element names for each species. U stands for genes of unknown location. The 5^th ^chromosome in Dpse (Element F) is not identified yet from the contigs and genes on it are likely included in the U group. The ragged distribution of the U group genes in Dpse is probably due to the mixed effect of F element genes and those on other elements. ANOVA indicates that element F genes (U group genes were used for Dpse) are significantly less biased and element A genes more biased with p < 10^-15 ^for all four species.

Figure 6 presents the cumulative distribution of codon adaptation index (CAI) for each of the six Muller-Sturtevant elements for four species for which good data are available on element linkage of available CDS. (Syntenic assignments at the time of the study were not sufficiently developed for the other eight species to perform this analysis.) Two patterns are apparent. First, element F CDSs have the least CUB; this is consistent with the more limited data presented previously [18,23] and shows that the predicted pattern expected in low-recombining regions is general at least for these four species. Second, as previously noted for Dmel [24](Singh et al. 2005), element A (X chromosome in all species) has the most biased CDS. Interestingly, in Dpse element D is also part of the metacentric X; this element does not differ significantly from the autosomes in CUB.

In the Additional File 1 and 2, graphs similar to those in Figure 6 are presented using ENC as the measure of CUB and the same conclusions are supported by this measure.

### Alternative codon usage

Given that mutation bias in *Drosophila* is generally toward A/T while CUB is generally toward G/C, the two forces, selection and mutation bias, are opposed. Also note that when selection is nonexistent or very weak, mutation bias will cause CUB but the pattern will be opposite that caused by selection. Thus non-directional measures of unevenness of codon usage such as ENC will identify genes/amino acids that have CUB due to both preferred GC in the wobble position (due largely to selection in *Drosophila*) and AT bias (due largely to mutation bias in *Drosophila*). Genes/amino acids with completely even codon usage would occur only when the mutation bias toward A/T is exactly balanced by selection toward G/C. The directional measure CAI does not have this property as it measures deviation from usage of a set of pre-defined preferred codons. Thus, unlike ENC, the CAI of genes/amino acids dominated by mutation bias would have a lower CAI than those with completely even usage of codons.

**Figure 7:**
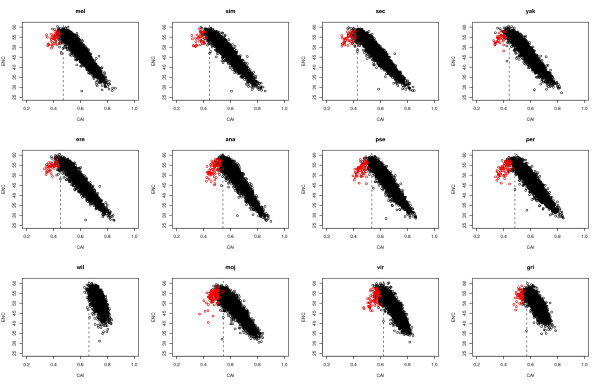
Scatter plot of CAI versus ENC values for each of the 12 genomes. The reference set for CAI is relative to each genome (see text for details). The red dots are the genes that are localized in the fourth chromosomes (element F) in *D. melanogaster*. The dashed line indicates the CAI index value that a gene with equal codon usage (no CUB) would have, which occurs when mutation bias to A/T is exactly balanced by selection to G/C. One interpretation of these graphs is that genes to the right of the dashed lines are dominated by selection for codon usage, whereas genes to the left are dominated by mutation bias.

Figure 7 presents the correlation between ENC and CAI for each of the 12 genomes. These two measures are quite tightly correlated, with all correlation coefficients associate with p < 10^-16^, and the correlation is quite linear over much of the range. If we assume that increasing CAI is largely controlled by selection for use of the preferred codon and ENC indicates bias that could be due to either selection or mutation bias, then the level of correlation between CAI and ENC may differ for different species depending on the relative balance of selection and mutation (likely associated with differences in effective population size as argued in the Discussion). The correlation coefficients in Figure 7 are all less than -0.8 except for Dwil (-0.51), Dvir (-0.75), and Dgri (-0.61). This pattern is consistent with the overall ENC of the different species (Figure 1) if we assume the scatter of the ENC measure is greater in these species due to the increased effect of mutation. That is, selection will cause a correlation between ENC and CAI, but the randomness of mutation will lessen this correlation when mutation bias becomes a relatively stronger force.

It is of considerable interest to note that the upper eight species in Figure 7, the evident linear association between ENC and CAI breaks down at the very lowest end of CAI on the left in these graphs. Genes at this end actually have somewhat lower ENCs resulting in a downward "hook" in this range of the relationship. This means they are biased when measured by unevenness of synonymous codon usage (ENC), but the direction of bias is away from usage of the optimal codon as defined by the reference set used to calculate CAI. As noted by the color differences, these genes tend to be those located on the non-recombining element F. This is as expected if mutation bias is dominating these genes so that they are biased (as measured by ENC) due to mutation bias toward A/T, opposite to usage of the optimal set of codons rich in G/C. The dotted line in each graph in Figure 7 are for completely equal usage of all codons, when mutation bias to A/T is exactly balanced by selection to G/C; thus one interpretation is that genes to the left of the dotted lines are dominated by mutation while those to the right are dominated by selection. [Note that this interpretation would only hold for cases as in *Drosophila* where mutation bias to A/T is opposite to selection for G/C, and thus this approach may not be generally applied to other organisms where this is not true.]

The four lower species in Figure 7 do not display much evidence for the downward hook associated with the other species, at least not associated with genes known to be on element F in Dmel. This is expected for Dwil, without a separate element F. The other species (Dmoj, Dvir, and Dgri) that do not exhibit this downward hook associated with element F genes are in subgenus *Drosophila*. Consistent with other evidence below, we speculate this is due to the fact that there has not been complete conservation of linkage of Dmel F element genes in subgenus *Drosophila*. If at least some genes that are found on element F in Dmel have now become incorporated in other chromosome arms, they would not be expected to produce the downward hook in Figure 7 evident in other species.

In the Additional File 1 and 2 we present graphs similar to those in Figure 7 identifying other genes with the alternative codon usage pattern. It is likely that many of these reside in other regions of the genome that have unusually low recombination such as near telomeres and centromeres.

Another way to identify alternative codon usage on the non-recombining F element is to compare the GC content at third positions of four-fold degenerate amino acids. Table 2 shows these data. An increase of 20–30% in A/T at these positions compared to the total data set is evident for most species. For Dwil, there is much less difference (6%) for these genes that have now become incorporated into normally recombining chromosome arms.

Finally we note the added evidence of the dominance of mutation bias on base composition of element F by examining the base composition of introns on this element compared to introns on all other elements. Table 2 shows that the A/T content of F element introns is consistently higher than introns on other elements. We suspect that the base content in non-recombining introns reflects most accurately the actual mutation bias which, fairly consistently across species, has a mean of 32% G/C, 68% A/T. This implies that introns in genes not on element F are still under some selective constraints (as indicated by a 35–41% G/C content) even after removing 50 bp at each end and eliminating predicted TE sequences. Note again that Dwil is the exception in that there is no difference in base composition between introns in genes that are on the F element in the other species and introns on all other elements (Table 2). This implies that the incorporation of these sequences into "normally" recombining chromosome arms subjects them to the same evolutionary forces as all other sequences.

Also evident in Table 2 is the fact that the F element genes in Dvir and Dgri have fairly high GC% at four-fold degenerate sites. This is consistent with the previous observation (Figure 7) indicating that there has not been complete conservation of synteny between Dmel and these two species in another subgenus diverged for about 50 million years.

### Regional patterns of base composition and its effect on codon usage

There is evidence that *Drosophila* are regionally heterogeneous in base composition [25,26]. Further Singh et al. [27] noted that substitution patterns are heterogeneous across the genome. Some evidence exists that this could be due to GC-bias-gene conversion associated with recombination [28,29]. It is not the intent of this paper to address this issue in detail, rather here we will simply address the evidence for such heterogeneity and how much effect it may have on CUB. To do this, we correlated the GC content of introns and with the GC content at the 3^rd ^position of 2-and 4-fold degenerate amino acids in the exons of the same gene. Table 3 displays the results; actual graphs are in the Additional File 1 and 2. As can be seen there are indeed significant positive correlations between GC content of introns and GC content of 3^rd ^position of 4-fold degenerate amino acids. The amount of variation in GC explained by this correlation (R^2^) is between 1 to 8%. These results are similar to those of Kliman and Hey [19] for only Dmel. So there is evidence for regional effects consistent with perhaps variation in recombination associated with Hill-Robertson effects or GC-biased gene conversion, although clearly the magnitude of the effect does not explain all, or even a majority of the variation in GC content at the 3^rd ^position. Because good information on genome variation in recombination is only known for Dmel and relatively little is known about the rates of GC-biased gene conversion, it is impossible to further speculate on this issue.

**Table 3:** Linear regressions of GC content of introns on coding sequences of the same gene for each species.^a^

	Coef^b^	p-value^c^	Rsq^d^	N^e^
mel	0.2460	2.52e-31	0.0412	3221
sim	0.1290	2.14e-07	0.0114	2344
sec	0.1570	1.62e-10	0.0169	2399
yak	0.1870	1.22e-13	0.0221	2463
ere	0.2460	1.86e-21	0.0364	2444
ana	0.2700	4.03e-26	0.0497	2195
pse	0.4140	9.82e-32	0.0708	1875
per	0.4210	1.05e-36	0.0802	1919
wil	0.2210	1.02e-21	0.0426	2109
vir	0.1780	3.58e-10	0.0160	2447
moj	0.1140	3.92e-05	0.0071	2374
gri	0.0588	2.42e-03	0.0034	2706

^a^GC content in coding sequences was calculated from the 3rd codon positions of 2- and 4-fold redundant amino acids.

^b^Multiplier coefficient of the linear model.

^c^The probability that the true coefficient value is different from zero.

^d^The fraction of total variability explained by the model.

^e^The number of genes used. Genes were chosen with the following criteria: from the homologous set we chose only genes that had at least one intron between 100 and 2000 bp in length after cleaning, and with at least 100 codons in the coding sequence.

## Discussion

### Stability and change in codon usage in *Drosophila*

As with many organisms, there is a characteristic pattern of codon usage in the genus *Drosophila*, at least for 11/12 species with complete sequenced genomes. With the exception of serine, all species have the same preferred codon for all amino acids (Figure 2 and Table 1). Codon usage for serine has a clear phylogenetic component in that all members of the *melanogaster *group have a different favored codon from all the other species. All favored codons in 11/12 species have either C or G in the third codon position.

*D. willistoni *is clearly the most different of all the species with regard to codon usage and is a clear exception to the generalities just expressed. The unusual codon usage in this species was first pointed out by [30] and has since been confirmed to be similar in all species in the lineage leading to Dwil including the *saltans *group [31-33]. Thus the shift in codon usage likely occurred ancestrally in the lineage leading to all extant members of this *willistoni-saltans *lineage. Generally, the change in codon usage in Dwil (Table 1) has been toward higher usage of T instead of C in the preferred codons (Arg, Leu, Gly, Asn, Asp, and Ile) or A for C (Pro). This results in a lower overall codon usage bias in this species as can be seen in Figures 1 and 5. But, interestingly not all amino acids shift in preferred codon, although there does seem to be an overall decrease in magnitude of CUB in Dwil even for those amino acids for which there is not a qualitative change in preferred codon.

This shift in codon usage could be due to a number of factors: (a) a quantitative increase in the level of mutation bias toward A/T, (b) a small effective population size so that the relative balance between mutation bias and selection has shifted toward the former even if the magnitude of mutation bias has not changed, and (c) change in the relative abundance of isoaccepting tRNAs so that the level and pattern of selection mediated by tRNA has shifted. There is some evidence that the magnitude of mutation bias toward A/T has increased in Dwil compared to the other species as assessed by intron base composition (Table 2). However, this magnitude of change in mutation bias by itself seems incapable of explaining changes in codon usage in Dwil [34]. Further, if we assume mutation bias is best reflected by base composition of the non-recombining introns of element F with a mean AT of 68% in 11/12 species, Dwil AT content for these same introns as well as all introns is marginally lower (66.5%) arguing that *neither the pattern nor strength of mutation bias in Dwil is significantly different from other species*. With regard to relaxed selection due to small population size, today Dwil has a very large distribution with dense populations [20,35,36] and its contemporary population size is undoubtedly very large. It is possible, however, that this may represent a relatively recent expansion since the Pleistocene and that the *willistoni *lineage went through a bottleneck that continues to affect patterns of molecular evolution. Consistent with this idea is the fact that Dwil has a higher level of non-synonymous polymorphisms than other species [37], implying relaxed selection. But again, this would seem to be insufficient to account for greater shifts in codon usage for some amino acids and not others. That the selective pressure for codon usage in the *willistoni/saltans *lineage has shifted, possibly due to a change in tRNA pools, is still a strong likelihood as argued in more detail in [33].

### Intensity of CUB and population sizes

As can be seen in Figure 1, there is considerable variation in level of CUB among the 12 species. Overall, this pattern is reasonably consistent with what can be inferred from the effective population sizes of the species, with selection for codon usage being more effective in larger populations; this is especially relevant as it has been shown that the level of selection on codon usage is very close to the border of when selection or drift dominate, l2N_e_sl ≅ 1 [38-42]. Dpse and Dper are widespread species in the western half of N. America and considerable data exist indicating high levels of gene flow among populations [e.g., [43,44]] consistent with a large N_e_. Also consistent with a large N_e _for Dpse is that it has a higher level of DNA plymorphism than Dmel or Dsim [20,45]. On the other hand, Dgri is an Hawaiian endemic with presumably a relatively small population and has the least degree of CUB (with the exception of Dwil). As first pointed out by [39] and now more thoroughly confirmed [[3]; Akashi et al. in preparation], Dsim has greater CUB than Dmel, again consistent with Dsim having greater molecular variation [45] indicating a larger N_e _than Dmel. Presumably, the relatively recent "out of Africa" history of the domestic populations of Dmel affected its historical effective population size, the effect of which can still be seen in the DNA patterns of variation. Dana has a history similar to Dmel in being a fairly recently domesticated species associated with human refuse. Among the other members of the *melanogaster *group, Dsec has the least CUB consistent with a small population size for an island endemic.

### Selection-mutation balance

Given that codon usage in *Drosophila* favors G and C in the wobble position of codons (Figure 2; Table 1) while mutation bias is toward A and T (Table 2), it is difficult to escape the conclusion that non-random synonymous codon usage is due, at least to a large degree, to selection. This is not to imply that selection need necessarily always act on synonymous codons nor equally on all genes. Figure 7 indicates a range of selection on genes with a minority having codon usage more indicative of mutation bias. Rather selection has acted on most genes over evolutionary time such that the footprint of selection is still evident in the newly available complete genome sequences. Also, as noted above, given that the selection coefficients associated with synonymous codon usage are near to the point where population size is crucial, given the inevitable fluctuations in populations over long periods of time, it seems very likely that the dominance of selection and mutation/drift has also fluctuated. In fact, one can see in the non-recombining element F genes, strong evidence that mutation and drift dominate. Given that we can document the expectations of mutation/drift dominance in non-recombining genes, makes it all the more evident that the patterns of DNA variation in "normally" recombining genes not on element F cannot solely be due to mutation/drift.

Also, we (Table 3) and others have noted that there may well be regional base composition effects that can affect codon usage. The precise quantitative interaction between factors influencing regional base composition and codon usage remains to be determined. However, the data presently available indicate that the influence exists, but it is not great.

But the level of selection must be weak as pointed out above, and different species, genes, and amino acids are differentially affected by selection. This explains the differences in Figures 2A and 2B. Figure 2A indicates that selection is acting reasonably consistently across all 12 species when considered on the entire gene level, yet Figure 2B indicates more complexity when considering individual amino acids for which the balance between selection and mutation bias may differ among species.

### Nature of selection

Two, interdependent, factors for selection on synonymous codon usage are speed and accuracy of translation. In unicellular organism (i.e., bacteria and yeast) it is well established that more highly expressed genes have greater CUB than lowly expressed genes and that the codons preferred are optimally translated by the most abundant isoaccepting tRNAs [46-49]. For *Drosophila*, similar observations have been made [e.g., [6-8]]. For example, there is a good correlation between level of expression and CUB for Dmel and Dpse for which microarray expression data are available [50]. In addition, experimental evidence indicates higher level of expression of alleles with optimal codons compared to non-optimal codons [51]. Finally, the relative abundances of isoaccepting tRNA in Dmel correlate with the preferred codons [7,52].

With regard to accuracy (avoidance of misincorporation of the wrong amino acid), it is known that synonymous codons can vary by as much as ten-fold in rates of misincorporation [53]. Akashi [54] presented evidence for selection for accuracy in *Drosophila* by showing that conserved amino acids among species have higher CUB than amino acids free to vary; the implication is that conserved amino acids are more crucial to protein function than those that vary among species. In this regard, it is interesting that among 2-fold redundant amino acids, Cys has the highest overall contribution to CUB and the second most overall all 18 amino acids (Figure 4) as well as being among the most "sensitive" to increasing gene CUB (Figure 5). Given its importance in three-dimensional structure of proteins by forming disulfide bridges, misincorporation at a Cys site should be strongly selected against. Leu has the greatest contribution on average to overall CUB (Figure 4). Leu is the most abundant hydrophobic amino acid [55] and thus may on average be more constrained against misincorporation than most amino acids. It is less clear why Lys and Gln are among the most sensitive two-fold degenerate amino acids (Figure 5), although generally (with the exception of Cys), A/G two-fold redundant amino acids are more biased in codon usage than C/T two-fold redundant amino acids.

Another potential explanation for different amino acids varying in intensity of CUB is that it is related to the number of different isoaccepting tRNAs for each amino acid. One possibility is that amino acids with one or very few tRNAs translating it are more prone to CUB than amino acids with several isoaccepting tRNAs. A comparison of the numbers of different isoaccepting tRNAs for each amino acid given in White et al. [56] and which amino acids contribute most or least to CUB does not obviously support this speculation.

The relative lack of codon bias for Asp can be more clearly associated with tRNA pools. Asp has both the least contribution of amino acids to the total CUB of a gene (Figure 4) as well as being the least sensitive (Figure 5). At least in Dmel (the only species studied for relative abundance of isoaccepting tRNAs), Asp is unique among amino acids as it is the only one for which the most abundant isoaccepting tRNA changes among developmental stages, with the most abundant tRNA in the larval stage optimally translating CAC and other stages optimally translating CAU [56]. Vicario [50] showed that genes with maximum expression at different developmental stages preferred Asp codon usage that matches the tRNA levels. The fact that genes expressed at different stages have selection for different Asp codons explains why this amino acid has relatively low contribution, on average, to overall gene CUB as well as why, when averaged over all genes (as done in Figure 5), Asp appears to respond slowly to increasing overall CUB.

If subsequent information such as levels of gene expression and tRNA pools becomes available for all 12 species considered here, then what we have referred to as "preferred" codons, may eventually become considered "optimal" codons *sensu *[6].

### Heterogeneity among chromosome arms (elements)

As noted in Figure 6, genes on the different chromosomal arms of *Drosophila*, or elements, have different codon usage. We discussed above the role of recombination and its lack in element F in many species of *Drosophila* and how this affects on codon usage. Less clear is why element A, an X chromosome in all species, has more biased CDS than other elements. This is true not only in *Drosophila*, but also in Caenorhabditis [34]. Two factors have been suggested, the hemizygosity of X in males and dosage compensation [34,50]. Exactly the role of these factors is unknown, although the evidence is that, whatever the factor(s) affecting X chromosome codon usage they are still weak. This is most evident in Dpse where an arm, element D, that is an autosome in many *Drosophila* has become part of a metacentric X chromosome. The codon usage bias in element D in Dpse is indistinguishable from other autosome arms (Figure 6), implying that not enough time has elapsed for this element to evolve a true X pattern of codon usage, although the incorporation of this element into the X likely occurred at least 10 million years ago [20]. It will be of considerable interest to observe what has occurred on element D in Dwil that evidently experienced a similar fusion of D and A elements around 25 million years ago [20]; this awaits syntenic assignments for this species.

### Prospects

Here we have presented largely descriptive aspects of codon usage in *Drosophila* based on the newly available complete genome sequences. It can be anticipated that with further analyses of these genomes, as well as acquisition of relevant data such as from microarray expression experiments for all 12 species, we will gain increased insights into the evolution of codon usage and its causes. Synonymous mutation, the basis of the evolution of codon usage, are clearly the kind of "nearly neutral" mutations that likely play a large role in molecular evolution [57]. Examination of codon usage and its evolution provides insights into the dynamics of this crucial class of mutations that have been fundamental in molding sequence patterns of genomes.

## Materials and methods

### Species

Complete (or nearly complete) genomic DNA sequences for twelve *Drosophila* species were announced by [3] and [4]. The species have a well-documented phylogenetic relationship as noted in Figure 1 of [3], and this figure can be consulted for details such as times of lineage splitting, chromosome composition, etc. The genus *Drosophila* is split into two major subgenera, Sophophora and *Drosophila*. Here, "*Drosophila*" will refer to the genus; the subgenus will be referred to as "subgenus *Drosophila*". These subgenera split from each other approximately 50 million years ago, and the available genomes are from 9 Sophophora and 3 subgenus *Drosophila* species. For ease of communication, we will use the following abbreviations to refer to the 12 species: *D. melanogaster*, Dmel; *D. simulans*, Dsim; *D sechellia*, Dsec; *D. yakuba*, Dyak; *D. erecta*, Dere; *D. ananassae*, Dana; *D. pseudoobscura*, Dpse; *D. persimilis*, Dper; *D. willistoni*, Dwil; *D. mojavensis*, Dmoj; *D. virilis*, Dvir; and *D. grimshawi*, Dgri.

### Data

Comparative Assembly Freeze 1 (CAF1) of all 12 genomes was downloaded from [58]. The final gene annotations for coding sequences (as of November 16, 2006) were available from [59]. We confined our analysis to the 6,698 protein-coding sequences (CDSs) that at this time have been identified as being homologous across all 12 species as defined in [60]. From these alignments we removed all codons from poorly aligned regions or those with insertion/deletions. This assured that we only compared codons for which there is strong evidence of homology across all 12 species. The *D. melanogaster *genome sequence was based on the Flybase release 4.3. There are 221 transcripts corresponding to 89 genes identified in the fourth chromosome of *D. melanogaster*. Thirty three of them were found in the 6,698 homologous CDS set.

### Codon usage analysis

Raw counts of the number of times a codon is used for each amino acid is the basic data. Various ways of summarizing codon usage have been proposed and used here.

### Relative synonymous codon usage, RSCU

The most straight forward way to measure codon usage bias is simply deviation from even usage. The relative synonymous codon usage (RSCU) statistic is calculated by dividing the observed usage of a codon by that expected if all codons were used equally frequently [61]. Thus an RSCU of 1 indicates a codon is used as expected by random (even) usage, RSCU > 1 indicates a codon used more frequently than expected randomly, and RSCU < 1 indicates a codon used less frequently than random.

### Effective number of codons, ENC

Another measure of CUB is "effective number of codons" [5] which we abbreviate ENC. This is also a measure of the unevenness of use of codons across all amino acids in a protein and is estimated by 2 + 9(1/F_2_) + (1/F_3_) + 5(1/F_4_) + 3(1/F_6_) where F_i _(i = 1,3, 4, 6). The value of F can be interpreted as the average "homozygosity" or probability of two randomly chosen codons for an amino acid being identical for the i-fold degenerate codon groups. If all codons for each amino acid are used equally (completely random usage), ENC will be 61; the other extreme would be if a single codon is used for each amino acid yielding an ENC of 20. Because the magnitude or strength of codon usage bias is negatively correlated with ENC, when correlating level of CUB with ENC, we use negative ENC. In its original formulation [5], ENC referred to the average "homozygosity" across a whole protein-coding sequence. Moriyama and Powell [7] modified it to be applicable to each amino acid, X, in a protein, called ENC-X. The maximum possible ENC-X is 2, 3, 4 or 6 depending on the degeneracy of the particular amino acid; thus to normalize codon bias among amino acids, ENC-X is scaled to range from 0 (no bias) to 1 (maximum bias); this is abbreviated sENC-X. The sum of sENC-X across all amino acids is denoted sENC for the protein. Note that sENC is positively correlated with the degree of CUB.

### Codon adaptation index, CAI

ENC, in its various formulations, and RSCU are non-directional measures of CUB being simply measures of unevenness. Codon adaptation index, CAI, was devised by Sharp and Li [62] as a directional measure of codon usage relative to a set of pre-defined reference optimal codons for a species. The identification of the reference set for each species analyzed here was done by examining the genes with the lowest ENC (highest bias) and accumulating at least 100 codons for each amino acid; depending on species, this involved between 12 and 20 most biased genes. Once the reference set is defined, a gene's CAI is simply CAI_obs_/CAI_max _where CAI_obs _is the geometric mean of observed RSCU across all amino acids of a protein and CAI_max _is the geometric mean of the maximum RSCU for each amino acid in the reference set and is the maximum possible given the particular amino acid composition of the protein being considered. Thus, CAI measures deviation from the optimum codon usage pattern defined for that species, 0 being furthest from the optimal set (no optimal codons used), and 1 indicating only usage of optimal codons.

#### Intron base composition

Intron sequences were identified from each of the 12 species' genomes based on the gene model GFF3 files available from the AAAwiki site cited above. In order to exclude possible transposable element (TE) sequences embedded in introns, we used the TE annotations based on the BLASTEr/tblastx analysis by Quesneville et al. [63] (GFF3 files available at [64]). All 12 genomes were first double masked against their BDGP TE and PILER-DF annotations. Next all introns from the 6,698 homologous gene set were extracted from each genome. Finally 50 bp each at the start and end regions of each intron were excluded to remove possible sites under splicing restrictions. This last step limited our intron analysis for those longer than 200 bp (100 bp after removal). In order to further exclude long introns potentially with embedded CDSs or with misidentified exons, we also examined introns that are longer than 100 bp and shorter than 2000 bp (after removing possible TE sequences and 50 bp from each end). The GC content of each intron was calculated and their unweighted average (disregarding intron length) was obtained from each genome. We also calculated the weighted GC content from the cumulative GC content from all introns concatenated. Further details of the intron data including the GC contents obtained with and without length limitations are available in the Additional File 1 and 2. For correlation analysis of GC contents between introns and CDS of the same gene (Table 2 and Additional File 1 and 2, only the first intron from each gene was used for the convenience.

## Authors' contributions

Saverio Vicario was responsible for analyzing all the coding sequence data, its interpretation, preparation of tables and figures, and contributed to writing the manuscript. Etsuko Moriyama was responsible for analyzing all the intron data, preparing the tables for these data, and contributed to interpretation of the results and writing of the manuscript. Jeffrey Powell guided the project, obtained financial support, and contributed to interpretation of the data and writing the manuscript.

## Supplementary Material

Additional file 1 Supplementary information for codon usage data from the 12 *Drosophila* genomes. Figures provided show the relative synonymous codon usage (Fig S1), bootstrap analysis of the preferred codons (Fig S2), sensitivity analysis of 2-fold degenerate amino acids (Fig S3), cumulative distribution of ENC for each chromosomal arm (Fig S4), scatter plot of CAI *versus *ENC (Fig S5), and fractional use of GC in introns *versus *exons (Fig S6). Click here for file

Additional file 2 Supplementary information for intron data from the 12 *Drosophila* genomes. The tables show detailed statistics for the intron data collected from each genome before (Table S1) and after (Table S2) filtering out possibly non-neutral regions. Click here for file
