# Supplementary material for: Codon usage in twelve species of Drosophila
Source: BMC Evol Biol. 2007 Nov 15;7:226. doi: 10.1186/1471-2148-7-226 (PMC2213667; doi:10.1186/1471-2148-7-226)

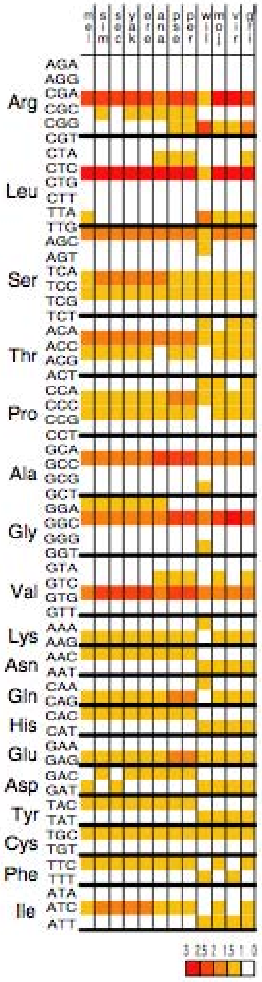

Fig S1. The relative synonymous codon usage (RSCU) for the 12 Drosophila species based on the all CDSs used. The colored shading indicates the RSCU values.

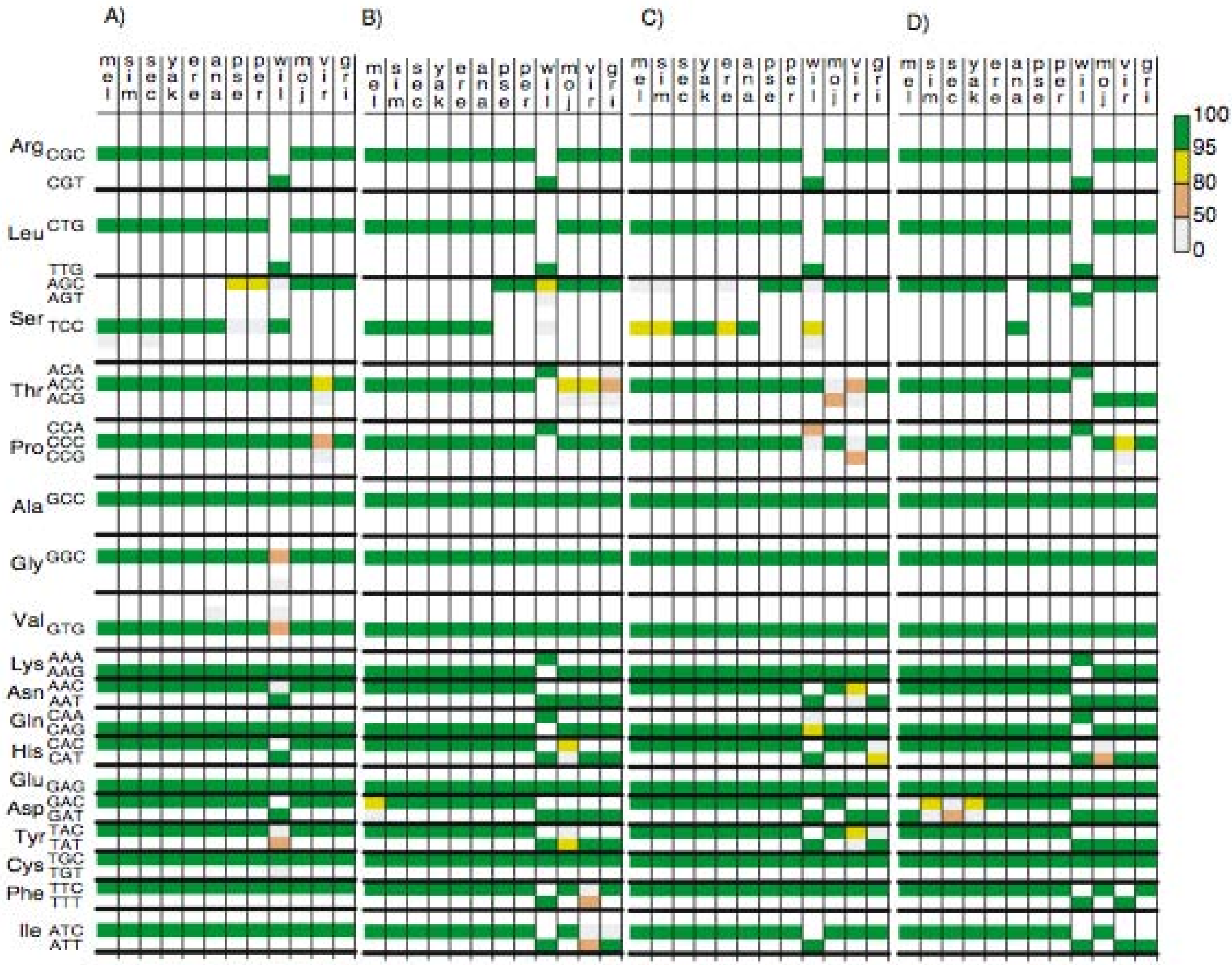

Fig S2. Bootstrap analysis for the three methods of identifying optimal codons. For each amino acid, only the codons that are identified as optimal for at least one species is shown. Bootstrap analysis was done for four different statistics: (A) the correlation between codon frequency and ENC, (B) the correlation between codon frequency and each amino acid codon usage bias (sENC-X), (C) RSCU based on the 10% highest biased genes, and (D) RSCU based on the all CDSs used. Bootstrap values (%) are shown in different colored shading as givein in the scale. See Matrials and Methods and Figure 2 for the details.

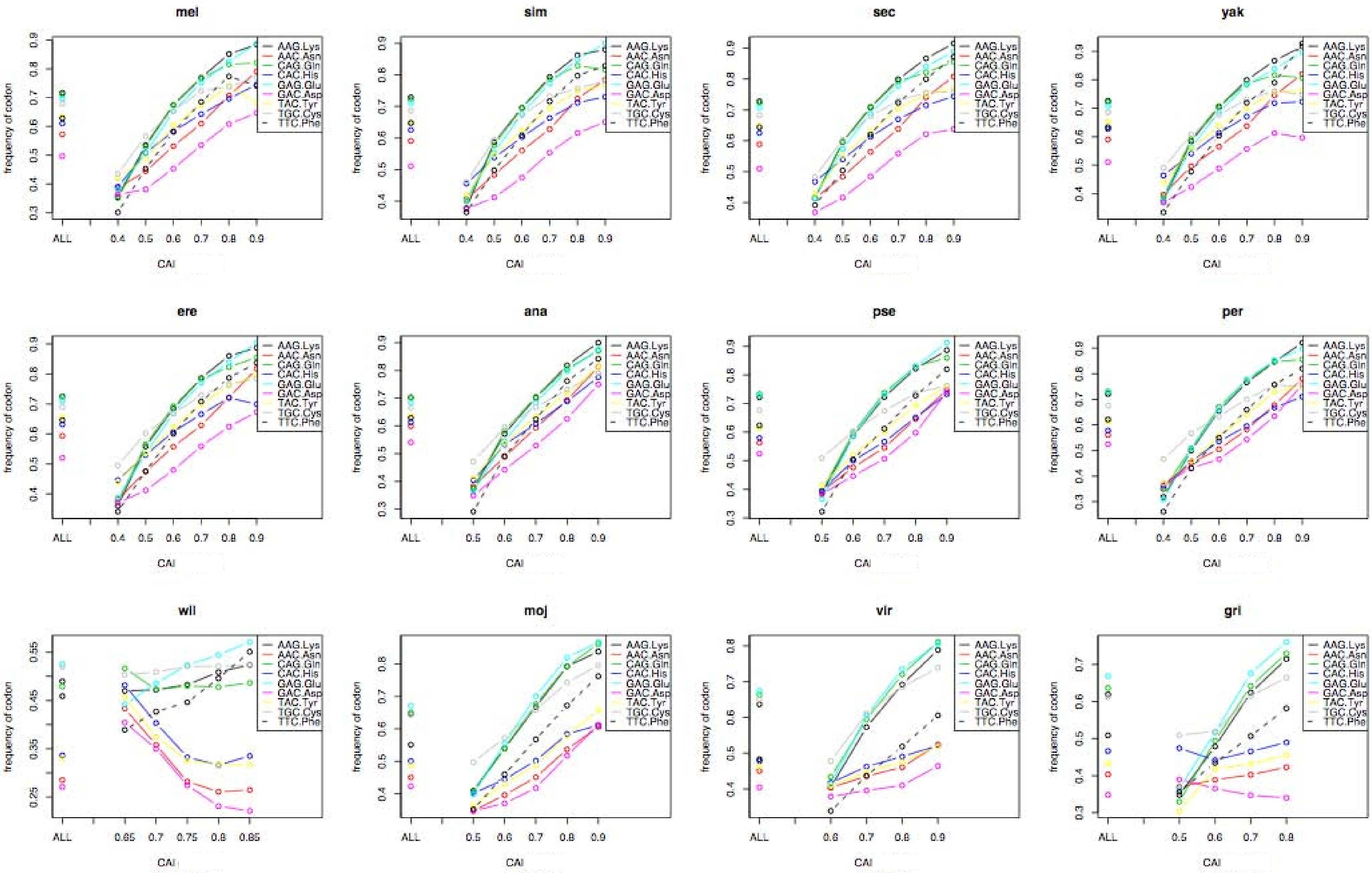

Fig S3. Sensitivity analysis of two-fold degenerate amino acids. Genes were ranked from low to high CAI and binned by 0.1 along the X axis. The mean frequencies of G or C-ending codons are plotted against CAI using the upper limit value of each bin. Circles to the left (above 'ALL') are the mean frequencies of codons for all CDSs used.

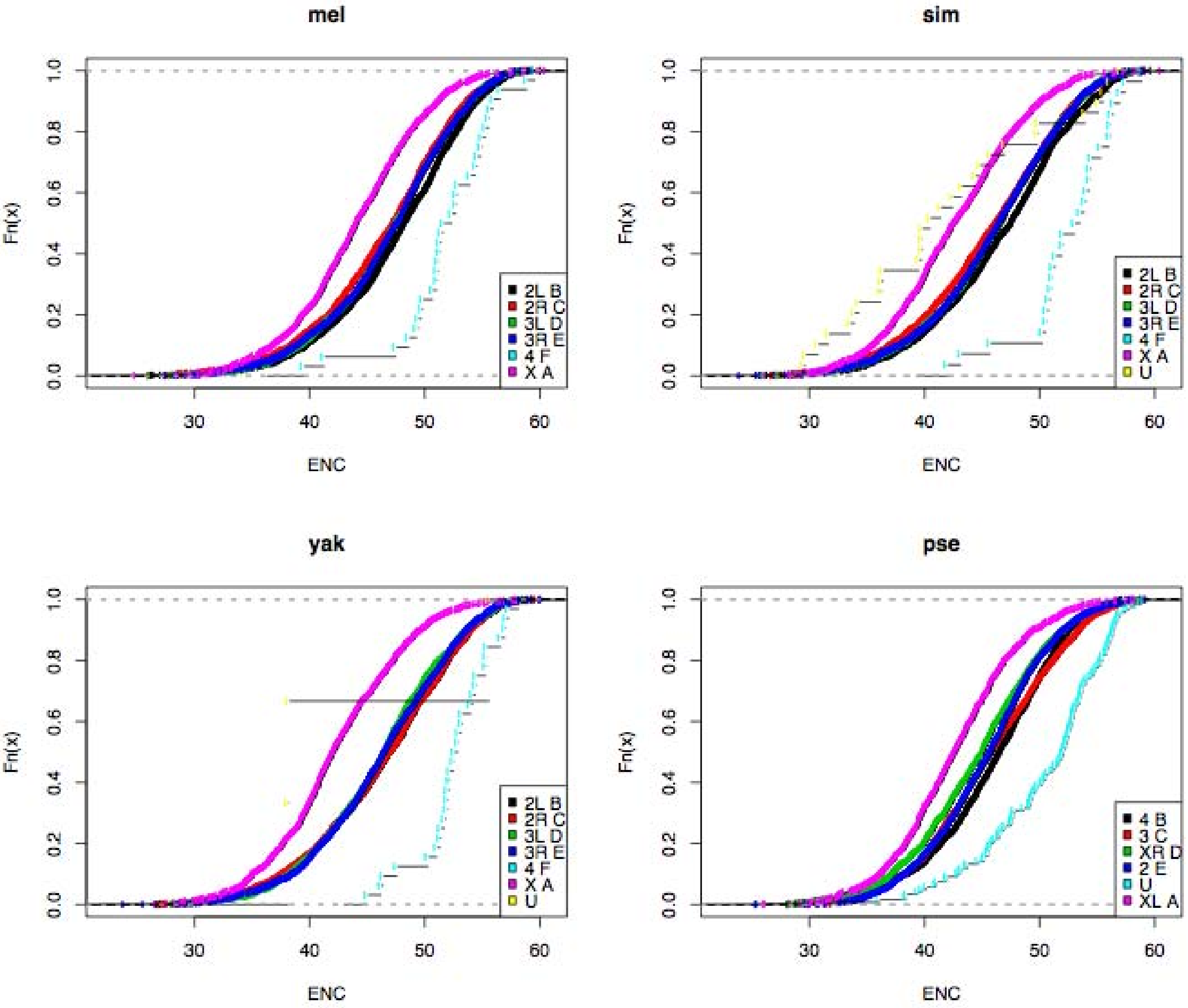

Fig S4. Cumulative distribution of ENC for each chromosomal arm. Each chromosomal arm and corresponding Muller/Sturtevant element is color coded and listed in each panel. U stands for genes of unknown location. The 5th chromosome in *D. pseudoobscura* is not identified yet from the contigs and gene on this chromosome are likely included in the U group. See Figure 6 for the plots using CAI.

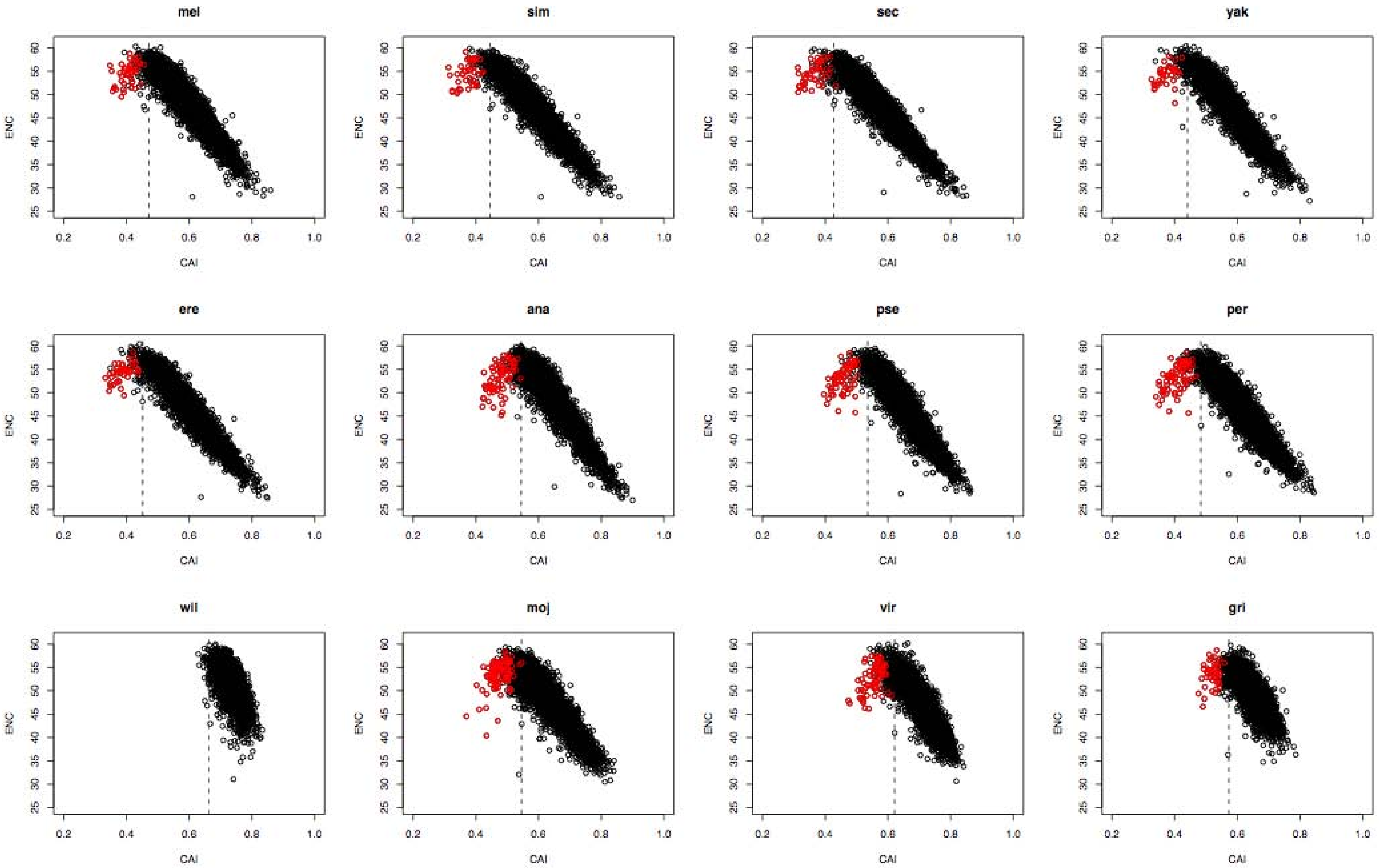

Fig S5. Scatter plot of CAI versus ENC values for each of the 12 genomes. The dashed line indicates the value of CAI for a gene with equal codon usage. Plots are the same as Figure 7, but with red dots showing genes whose CAI values are significantly lower than those with even usage (the dashed line) as estimated with a multinomial test with bonferroni correction for alpha = 0.05.

Figure S6. Fractional use of GC in introns versus exons for each species. The estimates of GC content are the same used for table 3 in the article. The mid line indicates identical usage between introns and exons. Very few genes are under the line (less GC content in exon than intron), while the vast majority is well over the line (larger GC content in exon than intron).

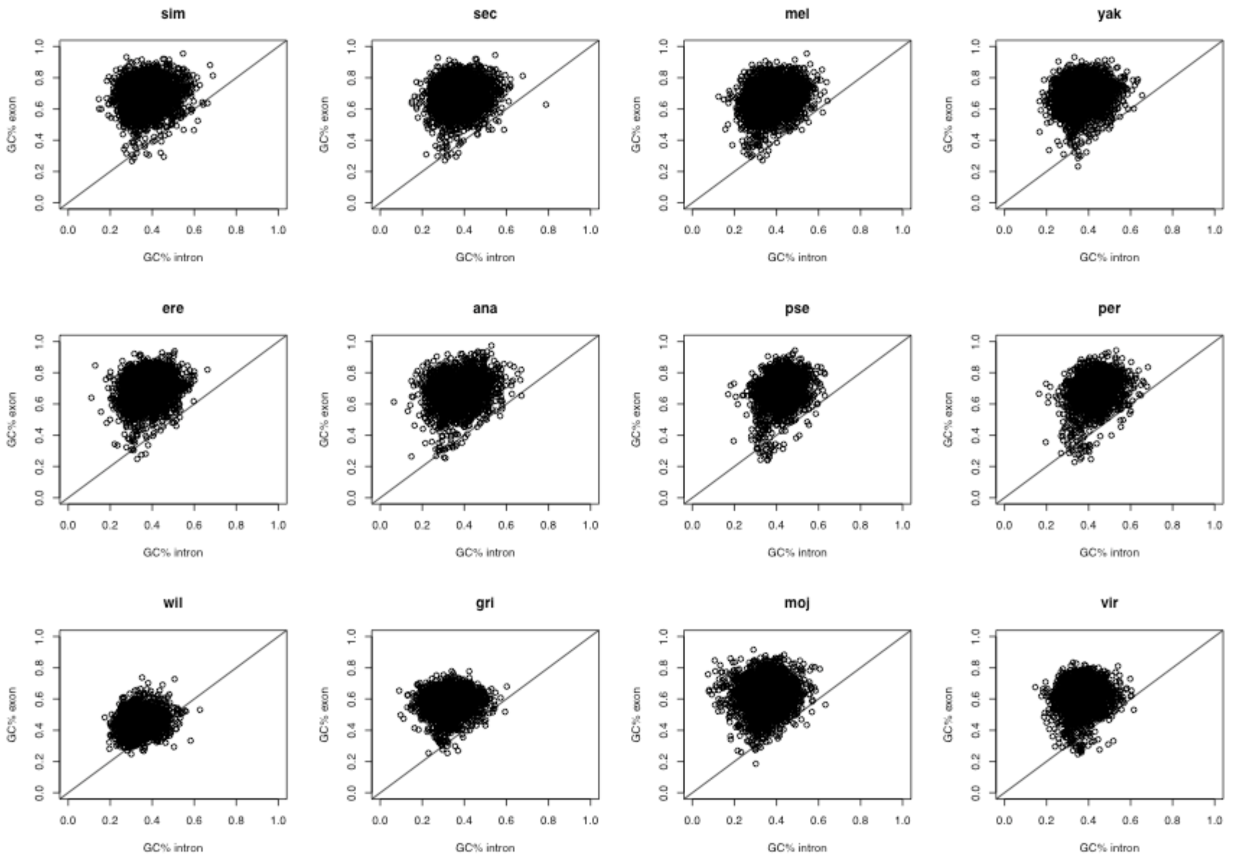

Supplement: Additional file 1 — Supplementary information for codon usage data from the 12 Drosophila genomes. Figures provided show the relative synonymous codon usage (Fig S1), bootstrap analysis of the preferred codons (Fig S2), sensitivity analysis of 2-fold degenerate amino acids (Fig S3), cumulative distribution of ENC for each chromosomal arm (Fig S4), scatter plot of CAI versus ENC (Fig S5), and fractional use of GC in introns versus exons (Fig S6). [file 1471-2148-7-226-S1.pdf]
