# Supplementary material for: Codon usage in twelve species of Drosophila
Source: BMC Evol Biol. 2007 Nov 15;7:226. doi: 10.1186/1471-2148-7-226 (PMC2213667; doi:10.1186/1471-2148-7-226)
Supplement: Additional file 2 — Supplementary information for intron data from the 12 Drosophila genomes. The tables show detailed statistics for the intron data collected from each genome before (Table S1) and after (Table S2) filtering out possibly non-neutral regions. [file 1471-2148-7-226-S2.pdf]

Table S1. Supplementary information for intron data from the 12 Drosophila genomes.

	Dmel	Dsim	Dsec	Dyak	Dere	Dana	Dpse	Dper	Dwil	Dmoj	Dvir	Dgri
**[All CDSs]**	19,389	17,049	21,332	18,816	16,880	22,551	17,328	23,029	20,211	17,738	17,679	16,901
No. introns	50,980	39,076	43,726	44,511	42,102	46,630	42,378	46,290	43,129	42,140	42,495	42,513
No. introns (200/2000)*	12,880	8,961	10,293	10,498	9,799	10,897	7,693	9,673	8,674	9,529	10,328	11,312
Total length (bp)	58,587,969	31,023,716	34,518,908	37,499,025	35,136,800	49,971,844	33,348,609	41,494,389	52,574,815	44,720,149	46,259,090	40,001,651
Average length (bp)	1149.2	793.9	789.4	842.5	834.6	1071.7	786.9	896.4	1219.0	1061.2	1088.6	940.9
GCi% (Weighted)	40.1	40.8	40.8	40.6	40.9	40.5	43.8	44.0	35.8	37.7	38.8	36.8
GCi% (Average)	36.6	37.5	37.6	37.0	38.2	36.3	42.0	42.8	33.0	36.0	37.1	33.6
GCi% (Ave 200/2000)	37.6	38.5	38.7	38.3	38.8	39.6	42.7	44.0	34.9	35.9	37.3	33.5
**[6698 homologue set]**												
No. introns	26,828	19,890	20,742	20,768	20,716	20,826	20,492	20,467	20,535	20,784	20,772	20,876
No. introns (200/2000)*	6,796	4,430	4,536	4,644	4,630	4,139	3,389	3,427	3,787	4,373	4,656	5,249
Total length (bp)	31,095,540	13,714,573	14,036,525	14,856,026	14,591,253	18,215,738	14,448,474	15,266,127	20,399,598	19,244,841	18,992,434	17,193,706
Average length (bp)	1159.1	689.5	676.7	715.3	704.4	874.7	705.1	745.9	993.4	926.0	914.3	827.2
GCi% (Weighted)	40.2	40.7	40.5	40.3	41.0	40.1	43.7	43.9	35.0	37.2	38.4	36.4
GCi% (Average)	37.0	37.7	37.4	36.7	38.1	34.9	41.7	42.2	32.3	35.1	36.7	33.2
GCi% (Ave 200/2000)	37.8	38.2	37.8	37.5	38.3	37.4	42.3	42.6	33.2	34.6	36.2	32.8
**[6665 non-4th chr homologue set]**												
No. introns	26,516	19,764	20,566	20,587	20,535	20,662	20,318	20,284	20,341	20,597	20,586	20,600
No. introns (200/2000)*	6,671	4,386	4,475	4,587	4,570	4,123	3,355	3,387	3,747	4,313	4,597	5,176
Total length (bp)	30,718,572	13,594,527	13,860,615	14,629,555	14,358,572	15,569,291	14,283,251	15,089,082	20,145,720	19,011,741	18,791,393	17,045,969
Average length (bp)	1158.5	687.8	674.0	710.6	699.2	753.5	703.0	743.9	990.4	923.0	912.8	827.5
GCi% (Weighted)	40.3	40.8	40.6	40.4	41.1	40.2	43.8	44.0	35.0	37.2	38.5	36.5
GCi% (Average)	37.0	37.7	37.4	36.8	38.2	34.9	41.8	42.3	32.3	35.2	36.7	33.3
GCi% (Ave 200/2000)	37.9	38.2	37.9	37.5	38.4	37.4	42.3	42.7	33.2	34.6	36.3	32.9
**[33 4th chr homologue set]**												
No. introns	312	126	176	181	181	164	174	183	194	187	186	186
No. introns (200/2000)*	125	44	61	57	60	16	34	40	40	60	59	73
Total length (bp)	376,968	120,046	175,910	226,471	232,681	2,646,447	165,223	177,045	253,878	233,100	201,041	147,737
Average length (bp)	1208.2	952.8	999.5	1251.2	1285.5	16136.9	949.6	967.5	1308.7	1246.5	1080.9	794.3
GCi% (Weighted)	32.1	34.0	32.3	33.3	32.6	39.4	35.4	35.9	35.8	35.8	34.2	30.2
GCi% (Average)	28.8	29.0	28.3	28.5	27.9	28.8	31.3	31.1	34.1	31.2	30.6	26.9
GCi% (Ave 200/2000)	30.4	32.1	30.7	31.2	30.4	28.9	33.5	33.5	33.1	34.6	32.4	26.3
**[All Dmel 4th chr genes]**	79											
No. introns	822											
No. introns (200/2000)*	353											
Total length (bp)	871,947											
Average length (bp)	1060.8											
GCi% (Weighted)	32.2											
GCi% (Average)	28.6											
GCi% (Ave 200/2000)	30.4											

*No. introns used after removing too short (< 200bp) and too long (> 2000bp) introns.
GCi% (Weighted): Cumulative GC% calculated from all introns concatenated
GCi% (Average): Average GC% calculated from all introns
GCi% (Ave 200/2000): Average GC% calculated from introns between 200bp and 2000bp

Table S2. Supplementary information for intron data from the 12 Drosophila genomes after removing transposable element seqeunces and 50bp each of start and end regions.

	Dmel	Dsim	Dsec	Dyak	Dere	Dana	Dpse	Dper	Dwil	Dmoj	Dvir	Dgri
**[All CDSs after removing TEs and 50 bp each from sart/end regions]**												
No. introns	47,676	39,076	43,726	44,511	42,102	46,630	42,378	46,290	43,129	42,140	42,495	42,513
No. introns (200/2000)*	12,301	8,949	10,113	14,020	9,717	10,111	7,596	8,988	8,454	9,448	10,202	11,320
Total length (bp)	48,919,379	27,100,612	29,239,690	34,005,517	29,499,000	36,104,413	28,845,315	34,345,799	44,256,343	38,671,234	38,227,733	35,887,276
Average length (bp)	1026.1	693.5	668.7	1636.8	764.0	774.3	680.7	742.0	1026.1	917.7	899.6	844.2
GCi% (Weighted)	40.3	41.1	40.9	40.7	41.3	40.4	44.0	44.2	35.1	37.3	38.5	36.7
GCi% (Average)	36.9	38.4	38.1	37.6	38.5	38.1	43.1	43.7	34.1	35.5	37.2	33.6
GCi% (Ave 200/2000)	37.5	37.8	37.4	37.1	37.8	37.5	42.4	42.8	33.3	34.5	36.4	32.7
**[6698 homologue set after removing TEs and 50 bp each from sart/end regions]**												
No. introns	24,960	19,890	20,742	20,768	20,716	20,826	20,492	20,467	20,535	20,784	20,772	20,876
No. introns (200/2000)*	8,769	4,505	4,606	4,700	4,698	4,227	3,460	3,509	3,860	4,472	4,769	5,324
Total length (bp)	27,923,159	12,046,139	12,223,018	13,238,523	12,842,331	14,447,517	12,679,626	13,379,761	18,175,927	17,016,748	16,473,328	15,410,088
Average length (bp)	1118.9	1545.96	1520.09	1636.81	1581.76	1990.56	2074.55	2107.05	2564.69	2236.1	2120.39	1753.54
GCi% (Weighted)	40.3	41.1	40.9	40.7	41.3	40.4	44.0	44.2	35.1	37.3	38.5	36.7
GCi% (Average)	36.9	38.4	38.1	37.6	38.5	38.1	43.1	43.7	34.1	35.5	37.2	33.6
GCi% (Ave 200/2000)	37.5	37.8	37.4	37.1	37.8	37.5	42.4	42.8	33.3	34.5	36.4	32.7
**[6665 non-4th chr homologue set after removing TEs and 50 bp each from sart/end regions]**												
No. introns	24,679	19,764	20,566	20,587	20,535	20,662	20,318	20,284	20,341	20,597	20,586	20,600
No. introns (200/2000)*	8,621	4,458	4,538	4,643	4,632	4,204	3,420	3,467	3,818	4,408	4,708	5,250
Total length (bp)	27,610,339	11,945,019	12,077,763	13,027,204	12,660,291	13,218,280	12,556,698	13,253,322	17,949,353	16,850,507	16,306,659	15,288,299
Average length (bp)	1119.0	1547.5	1522.1	1632.1	1580.6	1845.9	2081.3	2115.5	2563.5	2245.2	2127.1	1760.9
GCi% (Weighted)	40.3	41.1	41.0	40.8	41.4	40.8	44.1	44.3	35.1	37.3	38.6	36.8
GCi% (Average)	37.0	38.5	38.2	37.7	38.6	38.2	43.3	43.9	34.2	35.5	37.3	33.7
GCi% (Ave 200/2000)	37.6	37.9	37.5	37.2	37.9	37.5	42.5	43.0	33.5	34.5	36.5	32.8
**[33 4th chr homologue set after removing TEs and 50 bp each from sart/end regions]**												
No. introns	281	126	176	181	181	164	174	183	194	187	186	186
No. introns (200/2000)*	148	47	68	57	66	23	40	42	42	64	61	74
Total length (bp)	312,820	101,120	145,255	211,319	182,040	1,229,237	122,928	126,439	226,574	166,241	166,669	121,789
Average length (bp)	1113.2	1385.2	1370.3	1993.6	1670.1	12672.6	1556.1	1487.5	2665.6	1583.3	1618.2	1149.0
GCi% (Weighted)	32.1	34.0	32.3	33.3	32.6	39.4	35.4	35.9	35.8	35.8	34.2	30.2
GCi% (Average)	28.8	29.0	28.3	28.5	27.9	28.8	31.3	31.1	34.1	31.2	30.6	26.9
GCi% (Ave 200/2000)	29.8	32.7	31.4	32.0	30.5	30.6	33.8	34.2	33.3	33.9	32.9	26.5
**[All Dmel 4th chr genes]**	79											
No. introns	759											
No. introns (200/2000)*	396											
Total length (bp)	727,049											
Average length (bp)	957.9											
GCi% (Weighted)	31.3											
GCi% (Average)	28.4											
GCi% (Ave 200/2000)	29.8											

* No. introns used after removing too short (< 100bp) and too long (> 2000bp) introns.
GCi% (Weighted): Cumulative GC% calculated from all introns concatenated
GCi% (Average): Average GC% calculated from all introns
GCi% (Ave 200/2000): Average GC% calculated from introns between 200bp and 2000bp
